# Survey dataset on citizens’ perspective regarding government's use of social media for provision of quality information and citizens online political participation in Pakistan

**DOI:** 10.1016/j.dib.2020.106311

**Published:** 2020-09-12

**Authors:** Saman Arshad, Sobia Khurram

**Affiliations:** 1 Institute of Administrative Sciences, University of the Punjab, Quaid-e-Azam Campus, Lahore 54590, Pakistan; 2 Institute of Administrative Sciences, University of the Punjab, Quaid-e-Azam Campus, Lahore 54590, Pakistan

**Keywords:** E-governance, Social media, Online political participation, Pakistan

## Abstract

This article describes the survey-based dataset which was collected to assess citizens’ perspective regarding government's provision of quality information on social media and factors including transparency, trust, responsiveness of the agency, and citizens’ online political participation. The survey was conducted online through Google Forms and 388 responses were collected from the social media followers of a government agency in Pakistan. The questionnaire consisted of thirty-three pre-validated items derived from the existing literature. The data was collected using five-point Likert scale and is related to the extensive model tested in [1]. The analysis was performed in [1] by using AMOS 20. This article specifically highlights data relating to demographic variables (including gender, age, education level, employment status, and city) with citizen's online political participation and is analysed through SPSS. The data is valuable as it provides empirical evidence regarding the state of social media presence of a government agency in Pakistan and can be used by researchers to make inter-agency/cross-national comparisons. The dataset is available and can be accessed from Mendeley Data repository (https://data.mendeley.com/datasets/3mhk2jv94m/2)

## Specifications Table

**Table:** 

Subject	Social Sciences (General)
Specific subject area	E-governance: Social media in government
Type of data	Raw data in spreadsheet format (.xlsx) Tables
How data were acquired	The data was acquired by means of an e-survey on Google Forms by using both English and Urdu versions of the questionnaire. All questions in the e-survey were marked as mandatory to avoid missing values. The link to English version of the survey is as follows: https://forms.gle/Zx7stguXSg1DYU9a9
Data format	Raw Analysed
Parameters for data collection	The data for testing the relationship among constructs was collected using a five-point Likert scale. For measurement of all constructs, the five-point Likert scale followed the pattern of 1 = Strongly disagree, 2 = disagree, 3 = neutral, 4 = agree, 5 = strongly agree except for one construct (citizens’ online political participation) for which the scale followed the pattern of 1 = never, 2 = rarely, 3 = sometimes, 4 = often, 5 = always. The questionnaire also included questions related to demographics of the participants including gender, age, education level, employment status, and city.
Description of data collection	The Facebook and Twitter followers of a government agency in Pakistan (Punjab Food Authority) were contacted at random with the assistance of the social media team of the agency. The data collection process took the time duration of four months i.e. from April to August 2019 during which 388 responses were collected.
Data source location	Punjab, Pakistan
Data accessibility	Repository name: Mendeley Data identification number: http://dx.doi.org/10.17632/3mhk2jv94m.2 Direct URL to data: https://data.mendeley.com/datasets/3mhk2jv94m/2
Related research article	S. Arshad, S. Khurram. Can government's presence on social media stimulate citizens’ online political participation? Investigating the influence of transparency, trust, and responsiveness, Government Information Quarterly. 37(3) (2020), 101486. https://doi.org/10.1016/j.giq.2020.101486

## Value of the Data

• This dataset is useful as it provides citizens’ view regarding the quality information provided by government on social media and its influence on factors like transparency, trust, responsiveness and citizens’ online political participation in a developing country context where e-government is at an initial stage and thus there is scarcity of research in this area.
• The government institutions in developing countries can take benefit from this data by looking at impact of provision of quality information on important governance indicators like transparency, responsiveness and trust of citizens as well as online political participation and hence can formulate their communication policies and decisions based on this empirical evidence.
• This dataset can be used by the researchers to make comparisons between social media usage by different government institutions in developing countries or comparison of government institutions from developing and developed countries can also be made.
• This data represents primary data collected from a public agency on multiple relevant constructs measured through existing adapted tools which can be used by future researchers to compare across other study results using similar adopted tools.
• Future studies based on this data might aid to understand better how governments’ utilization of social media (especially developing counties) impact important constructs including transparency, responsiveness, trust, and online political participation.

## 1 Data Description

The survey data was collected by using a self-completion online questionnaire consisting of thirty-three items which were measured using five-point Likert scale. The Likert scale ranged from 1 = strongly disagree to 5 = strongly agree for all constructs except for the construct named online political participation for which it ranged from 1 = never to 5 = always. Other than these, five questions were asked related to the demographic characteristics of the respondents including gender (male, female), age (18–21, 22–25, 26–29, 30–33, 34–37, or Above 37), education level (matriculation/ O-Levels, intermediate/ A-Levels, graduation, post-graduation, or PhD), employment status (student, unemployed, employed/self-employed, or retired) and city. IBM SPSS version 20 was used to compile and analyse the data. In the Excel data file, the seven items coded as GPQI (GPQI1 to GPQI7) belong to the construct named government's provision of quality information on social media, the four items coded as PGT (PGT1 to PGT4) belong to the construct named as perceived government transparency, the four items coded as TGA (TGA1 to TGA4) belong to the construct named trust in government agency, the six items coded as PR (PR1 to PR6) belong to the construct of perceived government responsiveness, and the twelve items coded as PP (PP1 to PP12) belong to the construct named as citizens’ online political participation. PP was the dependent variable of the study.

Table 1 shows the descriptive statistics of PP across the two categories of gender. Table 2 shows the differences in the mean of PP across the two categories of gender. Levene's test revealed that there was homogeneity of variance (*p* > 0.05), therefore the assumption of running a *t*-test was fulfilled. T-test was used as gender had less than three categories. It can be seen from the table that there was no significant difference between male and female participants in terms of PP.

**Table 1:** Descriptive statistics (PP by Gender)

Gender	No. of cases	Mean (PP)	Standard deviation (PP)
Female	92	2.2455	.09108
Male	296	2.3820	.04967

**Table 2:** Independent sample t-test (Gender)

Levene's Test		T-test for equality of means				
F	*P*	t	df		Mean difference	*p*
.000	.994	-1.332		386	-.13657	.184

Table 3 shows the descriptive statistics of PP across the six categories of age. Table 4 shows mean difference between six categories of age in terms of PP. Levene's test revealed the absence of the homogeneity of variance (*p* < 0.05) which means that the assumption to run one-way analysis of variance (ANOVA) was violated. Therefore, instead of one-way ANOVA, the more robust tests i.e. Welch and Brown-Forsythe tests of equality of means were performed [2] which showed that there was a statistically significant difference between age groups in terms of PP (*p* < 0.05). To identify which of the groups showed statistically significant differences, the Games-Howell post-hoc test for multiple comparisons was performed as shown in Table 5, which further revealed that the differences between each group with another in terms of PP were not statistically significant.

**Table 3:** Descriptive statistics (Age by PP)

Age group	No. of cases	Mean (PP)	Standard deviation (PP)
18–21	41	2.6484	1.14238
22–25	109	2.4159	.90789
26–29	87	2.1216	.72278
30–33	71	2.3310	.79495
34–37	28	2.4732	.81620
Above 37	52	2.3157	.74979

**Table 4:** Test of equality of means (PP by Age)

Levene's Test				Welch Test		Brown-Forsythe Test	
F	df1	df2	*p*	F	*p*	F	*p*
3.807	5	382	.002	2.381	.042	2.422	.036

**Table 5:** Multiple comparisons (PP by Age)

(I) Age	(J) Age	Mean difference of PP (I-J)	*p*
18–21	22–25	.23247	.849
	26–29	.52673	.090
	30–33	.31739	.619
	34–37	.17516	.976
	Above 37	.33267	.594
22–25	18–21	-.23247	.849
	26–29	.29425	.122
	30–33	.08492	.986
	34–37	-.05731	.999
	Above 37	.10020	.977
26–29	18–21	-.52673	.090
	22–25	-.29425	.122
	30–33	-.20934	.524
	34–37	-.35157	.340
	Above 37	-.19406	.667
30–33	18–21	-.31739	.619
	22–25	-.08492	.986
	26–29	.20934	.524
	34–37	-.14223	.969
	Above 37	.01528	1.000
34–37	18–21	-.17516	.976
	22–25	.05731	.999
	26–29	.35157	.340
	30–33	.14223	.969
	Above 37	.15751	.957
Above 37	18–21	-.33267	.594
	22–25	-.10020	.977
	26–29	.19406	.667
	30–33	-.01528	1.000
	34–37	-.15751	.957

Table 6 shows the descriptive statistics of PP across five education levels. Table 7 shows the mean difference between five levels of education in terms of PP. Levene's test showed the presence of homogeneity of variance (*p* > 0.05) so the assumption to perform one-way ANOVA was fulfilled. The results of one-way ANOVA revealed that there was no statistically significant difference between groups of different education levels in terms of PP (*p* > 0.05).

**Table 6:** Descriptive statistics (PP by Education level)

Education level	No. of cases	Mean (PP)	Standard deviation (PP)
Matriculation/ O-Levels	43	2.2868	.97900
Intermediate/ A-Levels	63	2.5132	.91265
Graduation	142	2.3803	.83117
Post-graduation	137	2.2646	.82977
PhD.	3	2.2500	.36324

**Table 7:** One-way ANOVA (PP by Education level)

Levene's Test				ANOVA	
F	df1	df2	*p*	F	*p*
1.280	4	383	.227	1.017	.398

Table 8 shows the descriptive statistics of PP across four categories of employment status. Table 9 shows the mean difference between four categories of employment status in terms of PP. Levene's test revealed that the assumption about the homogeneity of variances was fulfilled (*p* > 0.05), therefore one-way ANOVA was performed. The results of one-way ANOVA show that there was no statistically significant difference between the categories of employment status in terms of PP (*p* > 0.05).

**Table 8:** Descriptive statistics (PP by Employment status)

Employment status	No. of cases	Mean (PP)	Standard deviation (PP)
Student	90	2.2880	.81899
Unemployed	75	2.2322	.80449
Employed/Self-employed	212	2.4072	.89905
Retired	11	2.5455	.74663

**Table 9:** One-way ANOVA (PP by Employment status)

Levene's Test				ANOVA	
F	df1	df2	*P*	F	*p*
.282	3	384	.838	1.129	.337

Table 10 shows the descriptive statistics of PP across the two categories of city (i.e. major cities/other smaller cities of Punjab). Table 11 shows the difference in the means of PP across the two categories of city. The Levene's test showed that the assumption about the existence of homogeneity of variance was fulfilled (*p* > 0.05), therefore, *t*-test was performed. The result of t-test reveals that there was a significant difference between means of two categories of city in terms of PP (*p* < 0.05). The mean of PP for the participants belonging to major cities of Punjab was slightly higher than the other cities of Punjab.

**Table 10:** Descriptive statistics (PP by City)

City	No. of cases	Mean (PP)	Standard deviation (PP)
Major cities	222	2.4242	.89613
Other smaller cities	166	2.2500	.80099

**Table 11:** Independent sample t-test (PP by City)

Levene's Test		T-test for equality of means			
F	*p*	T	df	Mean difference	*p*
3.692	.055	1.981	386	.17417	.048

## 2 Experimental design, materials and methods

For collecting the data, quantitative survey method was used which allows to collect data in a time- and cost-efficient manner and eliminates the chance of any bias and intervention of the researcher. The case of a single government agency i.e. Punjab Food Authority was chosen as it was found to be one of those agencies which were actively using social media platforms. The Facebook and Twitter followers of the agency constituted the population frame which were 318,454 as of April 09, 2019 with 317,812 Facebook followers and 642 Twitter followers which shows that Facebook was the majorly used and more important social media platform for the agency. The Yamane's formula [3] was used to reach the appropriate sample size (384) with sufficient statistical power to make inferences^1^, and so over the four months duration i.e. from April to August 2019, the social media followers of the agency were randomly contacted with the assistance of social media team of the agency as the researchers did not have direct access to the list of followers and 388 responses were collected. The link to the online survey was sent to social media followers of the agency as a private message on Facebook and Twitter. The online survey was uploaded on Google Forms. In the initial phase of data collection, the social media team of the agency highlighted the inability of some respondents to understand the survey in English language and therefore the researchers translated the survey in Urdu language as well as per the suggestion of the social media team of agency to augment the response rate. A separate Urdu language survey was created on Google Forms (https://forms.gle/58o8qk5oGUr3ozgN7) to collect data from those people who upon contact notified that they wanted to fill the survey in Urdu instead of English.

The data was automatically stored in separate spreadsheets for English and Urdu language questionnaires which was later compiled and transferred to SPSS 20 for analysis. All the items in the online survey were marked as mandatory to eliminate the chance of missing values.

## Ethics statement

The participation of the respondents in the data collection was voluntary without any coercion from researchers. In the beginning of the online survey, the informed consent of all participants was obtained. It was declared that the data was being collected for academic purposes only and that the participation of the respondents was entirely voluntary, and they were free to quit the survey at any stage. Moreover, it was also mentioned and was duly ensured that the anonymity of the participants was not breached at any stage of the study. Ethics approval for survey studies from local ethics committee was not required in this case as the study is based on an MPhil thesis and the concerned thesis supervisor approved the survey.

## Funding Organizations

None.

## CRediT authorship contribution statement

**Saman Arshad:** Conceptualization, Data curation, Visualization, Formal analysis, Investigation, Methodology, Resources, Software, Validation, Writing - original draft. **Sobia Khurram:** Conceptualization, Investigation, Methodology, Resources, Project administration, Supervision, Visualization, Writing - review & editing.

## Declaration of Competing Interest

The authors declare that they have no known competing financial interests or personal relationships which have, or could be perceived to have, influenced the work reported in this article.

## Appendix Supplementary materials

Image, application 1

## Footnotes

1 According to this formula, keeping the margin of error as 5%, the sufficient size of sample would be 384. The calculations involved in the Yamane/Slovin's formula are provided below: n = N / (1 + Ne^2^) Where, n = unknown sample size N = Population size e = Margin of error So, by putting the values of N as 318,454 and e as 0.05 (95% confidence interval) in the formula, we get:   n = 318,454 / (1 + (318,454 x (0.05)^2^)   n = 318,454 / 797.135   n = 384

## References

[1] Arshad S., Khurram S. (2020). Can government's presence on social media stimulate citizens’ online political participation? Investigating the influence of transparency, trust, and responsiveness. Gov. Inform. Q..

[2] Moder K. (2010). Alternatives to F-test in one-way ANOVA in case of heterogeneity of variances (a simulation study). Psycho. Test Asses. Model..

[3] Yamane T. (1967). Statistics, an Introductory Analysis.

